# Individual and population variation in isotopic niche between two sympatric cormorant species

**DOI:** 10.7717/peerj.20384

**Published:** 2026-01-12

**Authors:** Gabriela Piriz, Edwin J. Niklitschek, Valentina Mansilla Gamín, Karin Maldonado

**Affiliations:** 1 Programa de Doctorado en Ciencias mención Conservación y Manejo de Recursos Naturales, Universidad de Los Lagos, Puerto Montt, Los Lagos, Chile; 2 Centro i˜mar, Universidad de Los Lagos, Puerto Montt, Los Lagos, Chile; 3 Departamento de Ciencias, Facultad de Artes Liberales, Universidad Adolfo Ibáñez, Santiago, Chile

**Keywords:** Individual specialisation, Isotopic niche, Trophic ecology, Seabird coexistence, Seasonal variation, Sympatric cormorants

## Abstract

Coexistence among sympatric, functionally similar species often hinges on niche differentiation, especially as resource competition intensifies during the breeding season. Individual specialisation (IS) can promote coexistence by narrowing individual niches or increasing divergence among individuals. In colonial seabirds, aggregation at limited breeding sites and central-place foraging amplify both intra- and interspecific competition. Here, we assess seasonal shifts in individual and population isotopic niche widths in two sympatric cormorant species to elucidate the mechanisms underlying their coexistence. We analysed isotopic composition (*δ*^13^C and *δ*^15^N) in multiple-tissues to produce repeated measures within 111 individuals of red-legged cormorant (*Poikilocarbo gaimardi)* and imperial shag (*Leucocarbo atriceps)* captured on the Pirén Islet (Los Lagos, Chile) during breeding and non-breeding seasons. Multivariate generalised linear mixed models estimated isotopic niche components: total niche width (TNW), within-individual component (WIC), and between-individual component (BIC). We estimated IS (*i.e.*, the extent to which individuals exploit a narrower subset of the population niche) as BIC/TNW. *L. atriceps* exhibited 2.2-fold greater TNW than *P. gaimardi* during non-breeding and 2-fold greater during breeding. IS differed markedly between species: *L. atriceps* showed a higher IS during non-breeding (0.541 *vs* 0.213 in *P. gaimardi*), but decreased by 79.3% during breeding, whereas *P. gaimardi* increased IS by 52.1%. Niche width overlap was asymmetric and seasonally variable: *P. gaimardi* exhibited high overlap with *L. atriceps* (95.7% non-breeding, 89.6% breeding), whilst *L. atriceps* showed lower overlap (48.3% non-breeding, 43.7% breeding). Competition indices increased substantially during breeding in both species (305% in *L. atriceps*, 221% in *P. gaimardi*). Results suggest that coexistence relies on multiple mechanisms, including subtle population niche differentiation, contrasting IS between species, and divergent resource-use strategies. The high niche width overlap and narrower niche of *P. gaimardi* suggest greater competitive vulnerability for this Near Threatened species. Conservation of foraging habitat heterogeneity and prey availability is crucial for maintaining ecological opportunities that sustain these coexistence mechanisms.

## Introduction

Competition is a central force shaping both inter- and intraspecific niche segregation, fundamentally driven by the limitation of resources such as food, space, or breeding sites (Gause, 1934; Hutchinson, 1957; Tilman, 1982; Chesson, 2000). This pressure is expected to be particularly acute between populations of ecological specialists exhibiting large interspecific overlap between their trophic niches (Pianka, 1974; Connell, 1980; Letten, Ke & Fukami, 2017). Enhanced competition pressure may not only influences individual foraging strategies and population structure (Ward, Webster & Hart, 2006), but also drive evolutionary adaptations (Grant & Grant, 2006; Pfennig & Pfennig, 2012).

Classical niche theory (NT) posits that the coexistence of similar species requires niche differentiation to avoid competitive exclusion when resources are limited (Gause, 1934; Hardin, 1960; Hutchinson, 1957). Thus, shifts and/or contractions in diet, temporal patterns of activity, and spatial distribution all might serve to facilitate coexistence under increased competition (Schoener, 1974; Phillips et al., 2017). Nevertheless, Optimal Foraging Theory (OFT; Emlen, 1966; MacArthur & Pianka, 1966) predicts that intensified competition may be compensated by a broadening of the utilised resources, which leads to an expansion of the trophic niche and results in the emergence of a more generalist foraging population (Stephens & Krebs, 1986).

In cases where classical interspecific segregation is not evident, yet species continue to coexist, the mechanisms preventing competitive exclusion remain poorly understood. This may be in part because both NT and OFT have traditionally followed a population-level perspective, which overlooks the fact that entities within populations—such as sexes, age classes, contingents, or individuals—may use a relatively small and often consistent subset of the population niche. This intra-population niche partitioning has a great potential to reduce intra- and interspecific competition and to influence both population and community dynamics (Bolnick et al., 2011; Violle et al., 2012; Barabás & D’Andrea, 2016).

When intra-population niche partitioning occurs at the individual-level, it is termed individual specialisation (Bolnick et al., 2003). Following Bolnick et al. (2003)’s concept, individual specialisation is shaped by idiosyncratic factors, such as individual foraging experience, learned behaviours or phenotypical traits, such as the highly stereotyped capture techniques observed in marine predators (Woo et al., 2008), and the learned site-specific foraging behaviours observed in dolphin gulls (Masello et al., 2013). Hence, individual specialisation occurs regardless of other intra-population niche partitioning processes that may occur at multi-individual levels, such as between age-classes or sexes (*e.g.*, Cansse et al., 2024a), which may be also relevant for species coexistence.

Colonial nesting seabirds, concentrated in dense aggregations on a relatively small number of suitable breeding sites, face intensified intra- and interspecific competition that fundamentally shapes their foraging ecology (Ashmole, 1963; Wittenberger & Hunt, 1985). This spatial limitation amplifies competition for prey in adjacent waters and creates potential “halo effects” around colonies, whilst central-place foraging constraints during breeding force frequent returns to nests for chick provisioning (Birt et al., 1987; Gaston et al., 2007). Once released from the breeding duties, marine birds may follow divergent life-history strategies that include residency, dispersal, migration and partial migration, where only a segment of the population disperses or migrates seasonally (Newton, 2008; Bunnefeld et al., 2011; Amélineau et al., 2021).

Life-history strategies markedly affect ecological opportunity (*i.e.,* accessible resources and habitats) for species and groups that remain resident at their breeding sites compared to those that disperse or migrate away from such places (Araújo, Bolnick & Layman, 2011; Rueffler & Lehmann, 2024). During the non-breeding season, dispersive and migratory species or groups have the chance to exploit distant resources and habitats. Nonetheless, resident ones may benefit from reduced competition and seasonal immigration of alternative prey. These two contrasting sources of ecological variation—competition during central-place foraging and landscape-scale opportunity during dispersal—shape individual niche width and ultimately influence community structure and species interactions. Nonetheless, there is an unclear link between life-history types and either population or individual niche responses.

In some resident species and contingents, such as Kerguelen shags (*Phalacrocorax verrucosus;* Camprasse et al., 2017) or gentoo penguins (*Pygoscelis papua;* Herman et al., 2017), a pronounced increase in individual specialisation has been observed during the breeding season, when increased competition is expected due to central-place foraging constrains (Araújo, Bolnick & Layman, 2011). Nonetheless, the opposite response has been observed in other species, such as brown skuas (Krietsch et al., 2017) and Cory’s shearwaters (Zango et al., 2019), where individual specialisation has been found lower during breeding and higher during non-breeding seasons. Moreover, recent studies on black-faced cormorant show evidence of year-round individual specialisation in both resident and dispersive groups (Cansse et al., 2024a).

Examining competition and coexisting dynamics between closely related species is particularly valuable because they tend to share similar ecological demands, making competitive interactions more nuanced (Webb et al., 2002; Violle et al., 2011). For example, studies of closely related species, such as Darwin’s finches (Grant & Grant, 2006) and sympatric anurans (Cloyed & Eason, 2017), had revealed that even subtle differences (in morphology, timing, habitat use, and diet) can promote coexistence despite substantial interspecific niche overlap. Understanding these fine-scale niche partitioning mechanisms is essential for unravelling the processes that maintain species diversity and stability within ecological communities (Costa-Pereira et al., 2019).

The cormorant family (Phalacrocoracidae) provides an excellent model for investigating coexistence mechanisms, as its members share highly similar morphological and functional traits, often exhibiting significant ecological overlap (Frere, Quintana & Gandini, 2005; Grémillet et al., 2005; Frere et al., 2008). These generalist divers primarily feed on benthic and pelagic fish at high trophic levels, with several species coexisting in shared coastal breeding habitats and restricted foraging areas (Humphries, Hyndes & Potter, 1992; Sapoznikow & Quintana, 2003; Forero et al., 2004). While some previous studies have documented an important niche segregation between sympatric cormorants (Frere et al., 2008; Forero et al., 2004; Morgenthaler et al., 2025), others have found substantial niche overlap between coexisting members of this family (Sapoznikow & Quintana, 2003; Humphries, Hyndes & Potter, 1992).

Our study focuses on two sympatric cormorants: the imperial shag (*Leucocarbo atriceps,* classified as Least Concern), which disperses widely during non-breeding season (BirdLife International, 2018; Yorio et al., 2020), and the red-legged cormorant (*Poikilocarbo gaimardi,* listed as Near Threatened), which remains resident year-round (BirdLife International, 2021; Frere et al., 2004). Through stable isotope analysis, we quantified isotopic niche widths to assess seasonal variations in individual specialisation and niche width overlap between species at a major colony in southern Chile. Stable isotopes provide insights into both bionomic (resource-use) and scenopoetic (environmental) niche axes, with *δ*^15^N reflecting trophic levels and *δ*^1^^3^C indicating carbon sources (Bearhop et al., 2004; Cherel & Hobson, 2007; Newsome et al., 2007). Different tissue turnover rates reveal dietary information across annual stages, enhancing understanding of niche width in mobile marine organisms (Bearhop et al., 2006; Newsome et al., 2007; Jackson et al., 2011).

**Figure 1:**
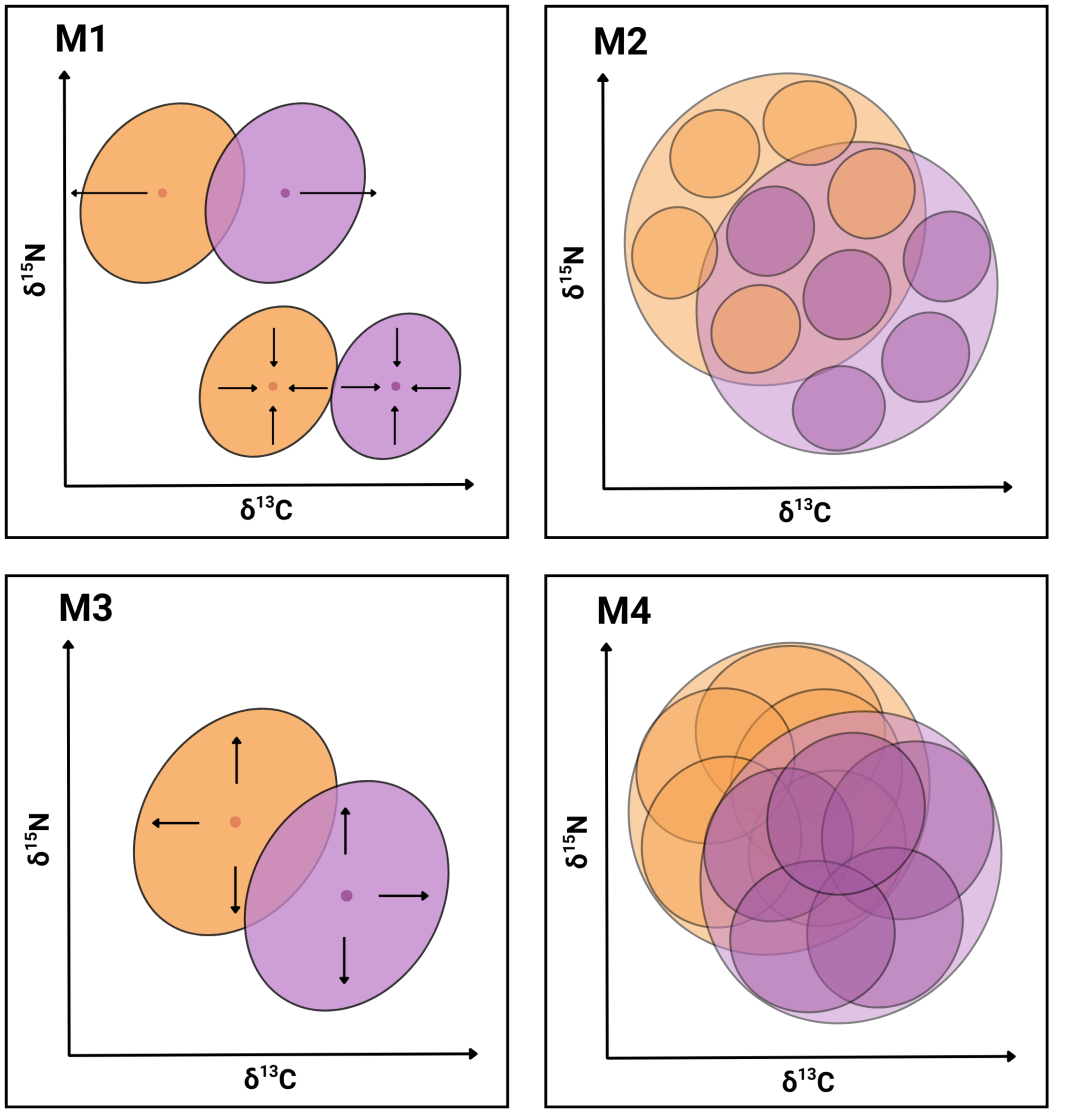
Conceptual graphical model illustrating four niche-based mechanisms facilitating coexistence in sympatric cormorants. Conceptual illustration summarising four mechanisms (M1–M4) hypothesised to facilitate species coexistence of two cormorant species. Each diagram depicts niches in isotopic bivariate space (*δ*^13^C, *δ*^15^N) and uses contrasting colours for the two species with transparent overlays for clarity. Outer ellipses represent the population-level niches of each species, whereas inner ellipses denote individual-level niches. **M1**: Segregation of population niches through increased distance between centroids (above) or contractions of population niches (below). **M2**: Enhanced individual specialisation due to reduced widths of individual-level niches and/or increased distance between individual-level niche centroids. **M3**: Expansion of the total population niche caused by the enlargement of population-level niches to exploit a broader resource spectrum. **M4**: Increased width and overlapping of individual-level niches, while keeping population-level niches stable.

In the present work, we seek evidence supporting the hypothesis that the coexistence of *P. gaimardi* and *L. atriceps* on the Pirén Islet is facilitated by one or more of the following mechanisms (Fig. 1): *Segregation of population niches* (M1), either through the contraction or shift in niche position to enhance partitioning of resources between species (*e.g.*, Morgenthaler et al., 2025; Forero et al., 2004); *Enhanced individual specialisation* (M2) through either a reduction of individual niche widths and/or an increase in distance between individual niche positions to enhance partitioning of resources between individuals (*e.g.*, Camprasse et al., 2017; Herman et al., 2017); *Expansion of the total population niche* (M3) towards a greater range of less profitable but relatively more abundant prey resources, which become exploited by different relatively specialised individuals (*e.g.*, Costa-Pereira et al., 2019); and *Expansion of the individual niche* (M4) toward a greater range of less profitable but relatively more abundant prey resources, which becomes exploited similarly by all population members (*e.g.*, Dehnhard et al., 2020). While the first two mechanisms are inspired by the NT (Gause, 1934; Hardin, 1960), the last two mechanisms are inspired by the OFT (MacArthur & Pianka, 1966; Stephens & Krebs, 1986).

Besides assessing data support to the four previously describe coexistence mechanisms, we wanted to reach a better understanding of the underlying ecological factors that might be triggering such responses. Thus, we produced and compared ecological opportunity and competition intensity proxies, both between seasons and between species.

## Methods

### Study site

Pirén Islet (41°42′S; 72°41′O) covers an approximate area of 3.15 hectares and is an important breeding site for numerous seabirds and marine mammals in the Reloncaví Sound, known for its high species richness and abundance (Cursach Valenzuela et al., 2022; Supplementary Material S1). This islet is not a protected area; however, a proposal is currently under review for the surrounding marine area to be recognised as a coastal marine space for indigenous peoples (ECMPO, for its acronym in Spanish). The islet features a land substrate at its centre covered with shrub vegetation and some trees, providing a resting and breeding ground for at least eight species of seabirds, including four cormorant species: *L. atriceps* (∼1,500 ind)*, P. gaimardi* (∼300 ind), Neotropic cormorant *(Nannopterum brasilianus,* ∼215 ind*),* and Magellanic cormorant *(L. magellanicus,* ∼50 ind*)* (Pérez-Santos et al., 2022). Recently, the reproduction of a fifth cormorant species, Peruvian booby cormorant (*L. bougainvillii*, ∼15 ind*)*, has been recorded, making this site the only location where all five cormorant species have been reported to breed along the SE Pacific coast.

The Reloncaví Sound is characterised by abundant freshwater, organic matter, and nutrient contributions from Andean rivers such as the Puelo, Petrohué, and Cochamó, which flow into it. These inputs enhance primary and secondary productivity, including that of zooplankton and small pelagic fish, and sustain a rich and diverse marine ecosystem (Iriarte, Pantoja & Daneri, 2014). The main factors that likely determine the seasonal variability of prey available for seabirds on the Pirén Islet are artisanal fishing, aquaculture production cycles (Jiménez et al., 2013), other anthropogenic activities in the coastal area (*e.g.*, agricultural run-off introducing nutrients and pollutants into nearshore waters) (Iriarte, González & Nahuelhual, 2010), climatic and oceanographic variability, and the seasonal migrations of prey species (Toledo et al., 2020; Heredia-Azuaje, Niklitschek & Sepúlveda, 2022). Additionally, the intense fishing activity in the surrounding waters has led to the overexploitation of small pelagic species (Subpesca, 2023), directly impacting piscivorous bird species.

### Capture and sample collection

Adult individuals of *L. atriceps* and *P. gaimardi* at the study site were sampled during the non-breeding season, defined as three-months season before the start of nesting activity (July–September) of 2022 (five sampling events), and the breeding season (chick-rearing, January–February) of 2022 and 2023 (six and two sampling events, respectively). Sampling was abruptly suspended in 2023 due to the highly pathogenic avian influenza epidemic that affected the SE Pacific coast (Azat et al., 2024). The study was conducted in accordance with the general principles of non-lethal sampling (Waugh & Monamy, 2016) and animal welfare (Zemanova, 2020). Necessary sampling permissions were obtained (Res. No 7850/2021, Servicio Agrícola y Ganadero, Chile), and the University of Los Lagos Scientific Ethics Committee approved sampling protocols (CEC-Ulagos ORD. 72/2021). A total of 111 individuals were captured: 25 *P. gaimardi* and 18 *L. atriceps* during the non-breeding season and 31 *P. gaimardi* and 37 *L. atriceps* during the breeding season.

Captures were conducted at night, employing two distinct methodologies based on light-attraction techniques. In some cases, researchers approached the island at night in a boat and attracted the cormorants using high-intensity lights (10,000–20,000 lumens), which exhibit positive phototaxis and dive toward the light source, landing in the water near the boat (King et al., 1994). These birds were then captured using nets and modified fishing rods (Morgenthaler, 2019; Alfaro & Aldabe, 2020). Alternatively, researchers landed on the island at night and employed spotlights to dazzle individual cormorants at close range. The intense illumination temporarily immobilises the birds through disorientation, allowing for direct capture with hand-held nets. Following capture, all birds were temporarily housed in ventilated cages (0.15–2 h maximum) before being measured and sampled on the boat. During the breeding season, only one adult per nest was captured, leaving the other free to guard the chicks. Handling lasted under ten minutes per bird, with no more than eight adults held at any time, thus minimizing disturbance and safeguarding chick welfare. After being measured and sampled, all individuals were banded before release to allow identification of recaptures and prevent repeated measurements within the same season.

A calliper was used to measure the length of the beak, head, tarsus, and wing, and a spring scale was used to determine body mass. Blood samples (0.5–1.5 ml) were obtained from the brachial or tarsal vein using heparinised syringes with 23–25 gauge needles and kept in ice until being processed at the laboratory.

Breast feathers and newly grown primary feathers (typically P6) were collected to obtain isotopic signatures from the non-breeding and breeding seasons, respectively. Since moult chronologies were not available for *P. gaimardi* and *L. atriceps*, the selection of these feather types to represent each season was based on published moult patterns for other *Leucocarbo* species (Rasmussen, 1987; Bernstein & Maxson, 1981), which matched our own field observations.

### Isotopic niche analysis

Since we were not able to obtain repeated measures from single individuals, we sample different tissues—plasma, red blood cells, and feathers—known to integrate dietary information over different temporal scales (plasma: 1–3 days, red blood cells: 3–4 weeks, feathers: whole molting period, (Bearhop et al., 2002; Hobson & Clark, 1992). Though this multi-tissue approach, we expected to capture both short-term and longer-term dietary variation across different life-history stages.

Once at the laboratory (4–8 h after extraction) blood samples were centrifuged for 20 min at 3,500 rpm to separate plasma and red blood cells. Both blood components were then dried (48 h at 60 °C) homogenised separately and encapsulated (∼1 mg) in tin capsules (Cherel et al., 2013). A 1-cm feather section was taken from the base of breast or newly grown primary feathers (confirmed by inspecting growth sheaths and feather condition), cleaned with alcohol and distilled water, dried (48 h at 60 °C), and cut into the tin cups to reach a nominal sample weigh of one mg.

The stable isotope analyses of carbon (*δ*^13^C) and nitrogen (*δ*^15^N) were conducted at the Centre for Stable Isotopes of the University of New Mexico, United States, using an elemental analyser coupled to an isotopic ratio mass spectrometer with a continuous-flow interface. The stable isotopes were expressed according to the following equation in *δ* (delta) notation (Coplen, 2011) in units per thousand (‰):

(1) \begin{eqnarray*}\delta X= \left( \frac{Rsample}{Rstandar} -1 \right) \ast 1000~\permil \end{eqnarray*}

where X is ^13^C or ^15^N, and R is the corresponding ratio of ^13^C:^12^C or ^15^N:^14^N. The standard value of R is based on Vienna Pee Dee Belemnite (VPDB) for ^13^C, and atmospheric nitrogen for ^15^N.

Following Ingram, Costa-Pereira & Araújo (2018), the repeated measures (*i.e.,* isotopic vales from multiple tissues) collected from each individual were used to produce explicit estimations of the within- and between-individual components (WIC and BIC, respectively) of the bivariate (two-dimensional) isotopic niche defined for each season and species (Ingram, Costa-Pereira & Araújo, 2018). WIC reflects the average individual resource width, which is, in turn, expected to represent the isotopic variability of the resources being exploited by each individual. At the same time, BIC quantifies the average degree of variation in resource use between individuals within a population (Roughgarden, 1972). Thus, the population total niche width (TNW) was calculated as the sum of both components (TNW = WIC + BIC; Roughgarden, 1972).

Although individual specialisation (IS) is traditionally defined as WIC/TNW, this relationship is counter-intuitive: lower values correspond to greater specialisation (Bolnick et al., 2002). To resolve this conceptual issue, Bolnick et al. (2007) proposed the V index (*V* = 1−IS = 1−WIC/TNW), which provides a more intuitive metric where higher values indicate greater specialisation. Following this logic, we adopted the metric IS = BIC/TNW (Rosenblatt et al., 2015; Bolnick et al., 2011), which similarly ensures that values directly reflect the intensity of specialisation. Thus, the degree of IS increases when individual niches are narrower (low WIC) and/or more segregated (high BIC).

Isotopic niche components (WIC, BIC) and derived metrics (TNW and IS) were estimated using Bayesian generalised linear mixed models (Hadfield, 2010), which modelled *δ*^1^^3^C and *δ*^15^N responses jointly, within a multivariate framework. Fixed effects included species, season (breeding and non-breeding), and sampling campaign, while individual identity (ID) was modelled as a random effect (Ingram, Costa-Pereira & Araújo, 2018). This random effects structure allowed partitioning the variance into between-individual (G-structure) and within-individual components (R-structure).

BIC and WIC metrics were finally computed as the sum of the G and R matrix eigenvalues, respectively, and were represented by ellipses defined at the 67% confidence level. This multivariate framework parallels Jackson et al. (2011)’s SIBER ellipses, using Bayesian estimation to quantify niche width, position, and overlap, while extending it, *via* variance–covariance partitioning, into within- and between-individual components (Ingram, Costa-Pereira & Araújo, 2018). As recommended by these authors, and due to the absence of species-specific tissue discrimination factors for our cormorant species, we standardised *δ*^1^^3^C and *δ*^15^N values within tissue types across species (z-score transformation: mean = 0, SD = 1) before estimating variance–covariance matrices. This standardisation eliminated systematic differences between tissues while maintaining the biologically relevant variations within-tissues and between species (Ingram, Costa-Pereira & Araújo, 2018).

Bayesian generalised linear mixed models were fit using the R package ‘MCMCglmm’ (Hadfield, 2010). Each MCMC chain was run for 100,000 iterations with a thinning interval of 10 and a burn-in of 5,000 iterations. Model convergence was assessed utilising the Gelman–Rubin diagnostic based on five independent runs (Gelman et al., 2014).

### Indicators of ecological opportunity

Although it was not possible to directly measure the magnitude of the main ecological factors believed to affect individual specialisation, we employed two indirect indicators previously proposed to evaluate ecological opportunity, defined as the diversity of exploitable resources accessible to a population (Ingram, Costa-Pereira & Araújo, 2018). Seasonal variation in ecological opportunity was indirectly assessed by analysing prey (fish otoliths, cephalopod jaws, polychaete remains, and crustacean exoskeletons) found in pellets collected on the island (Morgenthaler, 2019). Based on collection sites near *L. atriceps* nests and pellet morphology, most pellets likely originated from this species.

Sixty pellets were collected during the non-breeding season (June and September) and 70 during the breeding season (November and December) of the years 2020 and 2022. Pellets were wrapped in aluminium foil and kept frozen until thawed at 60 °C for 20 min before dissection. Distilled water was used to detach and separate the remains under a magnifying glass, which were then stored in Eppendorf tubes with 70% alcohol, except for the otoliths, which were stored dry. Each structure was photographed using magnifications of 0.5X and 6.3X. Identification was made using taxonomic keys from García-Godos (2001) and Xavier & Cherel (2021) and assistance from experts (P Toledo, B Pacheco, and H Heredia-Azuaje, Centro i˜mar, Universidad de Los Lagos). Nominal richness and the reciprocal Simpson index (Magurran, 2004) were used to characterise seasonal trends in prey richness and diversity.

### Indicators of competition

To quantify the intensity of competitive interactions in our study system, we estimated potential competition using the effective abundance of hetero and conspecifics (Nz; Costa-Pereira et al., 2018), a metric that integrates both the local abundance of competing species and their phylogenetic relatedness as a proxy for ecological similarity. This approach is grounded in the principle that competition intensity should be stronger between closely related species due to their shared evolutionary history and similar ecological requirements.

Competition indices were determined for each species during both seasons based on the presence of species from the orders Suliformes, Pelecaniformes, Charadriiformes, and Sphenisciformes, which constitute the primary marine bird taxa exploiting similar prey resources, particularly fish and cephalopods (Cherel & Klages, 1998; Furness et al., 2012). These taxonomic groups represent the main seabird competitors in marine ecosystems, sharing overlapping trophic niches and foraging habitats despite differences in foraging strategies and morphological adaptations (Forero et al., 2004; Dehnhard et al., 2020). While trophic overlap varies among taxa, our pellet analysis confirmed that cormorants exploit both benthic and pelagic prey, including small pelagic fish also targeted by other marine birds.

Phylogenetic distance weighting (α function, Eq. (2)) was used to ensure that closely related species contribute more strongly to competitive pressure, reflecting potential rather than direct competitive interactions. Census data were collected between May–August for the non-breeding season and October–January for the breeding season, derived from comprehensive surveys conducted between 2020 and 2024 using standardised visual counting techniques combining aerial observations with DJI Mavic Pro drone platforms and systematic direct counts using 10 × 42 binoculars, conducted during optimal weather conditions by trained observers following established protocols for colonial seabird monitoring (Bibby, 2000).

The effective abundance metric was based on the quantitative genetic competition model proposed by Doebeli (1996), which models competitive interactions as a function of phenotypic similarity between species. The index is calculated as:

(2) \begin{eqnarray*}Nz=\sum N(z{^{\prime}})\mathrm{\alpha }(z,z{^{\prime}})\end{eqnarray*}

where, N(z′) represented the observed absolute abundance of hetero and conspecifics, unlike Costa-Pereira et al. (2018), who employed relative abundance measures, we utilised absolute abundance values to avoid potential scaling artefacts. This distinction is important because relative abundance indices can produce identical values regardless of actual population sizes, potentially masking true differences in the intensity of competitive pressure.

The competitive impact α(z, z′) represents the per capita reduction in population growth of focal species z caused by each individual of competitor species z′ and therefore quantifies the combined effects of both intraspecific competition (when z = z′) and interspecific competition (when z≠z′) experienced by individuals of the focal species. This comprehensive competition index is modelled using a Gaussian decay function, such that competitive intensity declines with increasing niche (phylogenetic) dissimilarity (Doebeli, 1996; Costa-Pereira et al., 2018):

(3) \begin{eqnarray*}\mathrm{\alpha }(z,z{^{\prime}})=\exp \nolimits ( \frac{-(z-z{^{\prime}})^{2}}{2{\sigma }_{\mathrm{\alpha }}^{2}} )\end{eqnarray*}

where, the phenotypic difference (z,z′) corresponded to the phylogenetic distance between species pairs, calculated from a time-calibrated ultrametric phylogenetic tree integrating datasets from BirdTree.org (Jetz et al., 2012) with high-resolution cormorant phylogeny from Kennedy & Spencer (2014). The integrated tree was constructed using the R packages “ape” (Paradis & Schliep, 2019), “phytools” (Revell, 2012), and “picante” (Kembel et al., 2010).

Temporal scaling compatibility between phylogenies was achieved through proportional rescaling prior to integration using bind.tree() function, and ultrametricity was enforced using force.ultrametric() with non-negative least squares method. Final phylogenetic distances were normalised to 0–1 scale for statistical analyses. The parameter *σ*^2^ = 0.05 defines the rate at which competitive intensity declines with increasing phylogenetic divergence (Eq. (3)), following Costa-Pereira et al. (2018) to approximate the competitive function as a Gaussian curve.

### Statistical inference

We employed a multi-model inference approach (Burnham & Anderson, 2004), utilising the second-order Akaike’s Information Criterion (AICc) for model selection and the Akaike weight (AICw) to assess the probability of each model being the most informative one within a given set of competing alternatives (Burnham & Anderson, 2004). Generalised linear models (GLMs, McCullagh & Nelder, 1989) were used to evaluate interspecific differences in morphometric traits (mass, beak length and width, wing length and leg length) between species (single explanatory variable). The gamma distribution proved to be more informative (lower AICc) than the Gaussian one for these data.

Generalised linear mixed models (GLMMs, Breslow & Clayton, 1993) were used to examine univariate variation in *δ*^1^^3^C and *δ*^15^N between tissue types and species. Main effects and interactions of these explanatory variables were included as fixed effects. At the same time, individual ID and season were treated as random effects to account for repeated measures within individuals across tissues and seasons. Multi-model pairwise comparisons were then performed to assess (1) differences among tissue types within each species, and (2) differences between species within each tissue type.

Bayesian inference for testing alternative coexistence mechanisms was based directly on the posterior distributions of niche components and derived metrics (Gelman et al., 2014). Specifically, for M1 (population niche segregation), we assess the probability of directional differences in species niche overlap between seasons. Niche overlap, here, was calculated as the proportion of one species’ ellipse (TNW at 67%) that was overlapped by the other species’ ellipse, at each MCMC sample. For M2 (enhanced individual specialisation), we evaluated the probability of directional differences in IS (BIC/TNW ratios) within species between seasons. For M3 (population niche expansion), we assessed the probability of directional differences in TNW between seasons within species. Lastly, for M4 (individual niche expansion), we computed the probability of directional differences in WIC between seasons within species. Reported *p*-values corresponded to the posterior probabilities of selected comparisons evaluated at each MCMC sample (*e.g.*, P (TNW_*L*.*atriceps*_ >TNW_*P*.*gaimardi*_) = proportion of posterior samples where *L. atriceps* TNW exceeded *P. gaimardi* TNW).

Ecological opportunity was analysed using GLM to evaluate seasonal effects upon (i) nominal richness (Poisson distribution) and (ii) the inverse Simpson diversity index (Gaussian distribution), following Magurran (2004). Competition intensity was quantified using the effective abundance of competitors (Costa-Pereira et al., 2018), which integrates the local abundance and phylogenetic relatedness of seabird species present during surveys.

Distribution assumptions were tested using Hartig’s (2022) simulated residuals method and Kolmogorov–Smirnov tests. Homoscedasticity was assessed using Breusch-Pagan tests (1979). The lack of independence among sampling events was addressed through the use of mixed models (Searle, 1987). For morphometric and ecological opportunity analyses, model selection followed multi-model inference based on AICc and AICw (Burnham & Anderson, 2004).

All statistical analyses were conducted using R version 4.4.3 (R Core Team, 2025). GLMs and GLMMs were fitted using the R package ‘glmmTMB’ (Brooks et al., 2017). Marginal means and their standard errors were estimated *via* the R package ‘emmeans’ (Lenth et al., 2025). *Post-hoc* pairwise comparisons were performed using a customised version of the multiple comparison function ‘multComp’ (Hothorn et al., 2025), which we adapted to suit the specific requirements of our posterior analysis. Bayesian multivariate generalised linear mixed models were implemented using the MCMCglmm package (Hadfield, 2010). Overlap of species’ total niche width (TNW) ellipses was calculated employing the sf package (Pebesma & Bivand, 2023).

## Results

### Capture success and morphometric information

A total of 111 individuals were captured: 25 *P. gaimardi* and 18 *L. atriceps* during the non-breeding season and 31 *P. gaimardi* and 37 *L. atriceps* during the breeding season. On average, *L. atriceps* exhibited higher values in mass (1.36 fold), wing length (1.06 fold), beak breadth (1.24 fold), and leg length (1.13 fold) than *P. gaimardi*. In contrast, beak length was similar between species (Table S2).

### Isotopic signatures

The stable isotopes analysis revealed clear differences in *δ*^1^^3^C and *δ*^15^N between species and tissue types (Table 1). For *δ*^1^^3^C, body feathers exhibited similar values between species, while plasma showed the most depleted values of all tissues, with *L. atriceps* being lower than *P. gaimardi* (Table 1). Red blood cells exhibited intermediate *δ*^1^^3^C levels, also differing between species, with *L. atriceps* again showing values 2.47% lower than *P. gaimardi*. Regarding *δ*^15^N, interspecific differences were evident only in plasma, where *P. gaimardi* was notably enriched compared to *L. atriceps*. Primary feathers showed the highest *δ*^15^N enrichment in both species, whereas red blood cells exhibited the lowest values, particularly in *P. gaimardi* (Table 1).

### Niche components and individual specialisation

**Figure 2:**
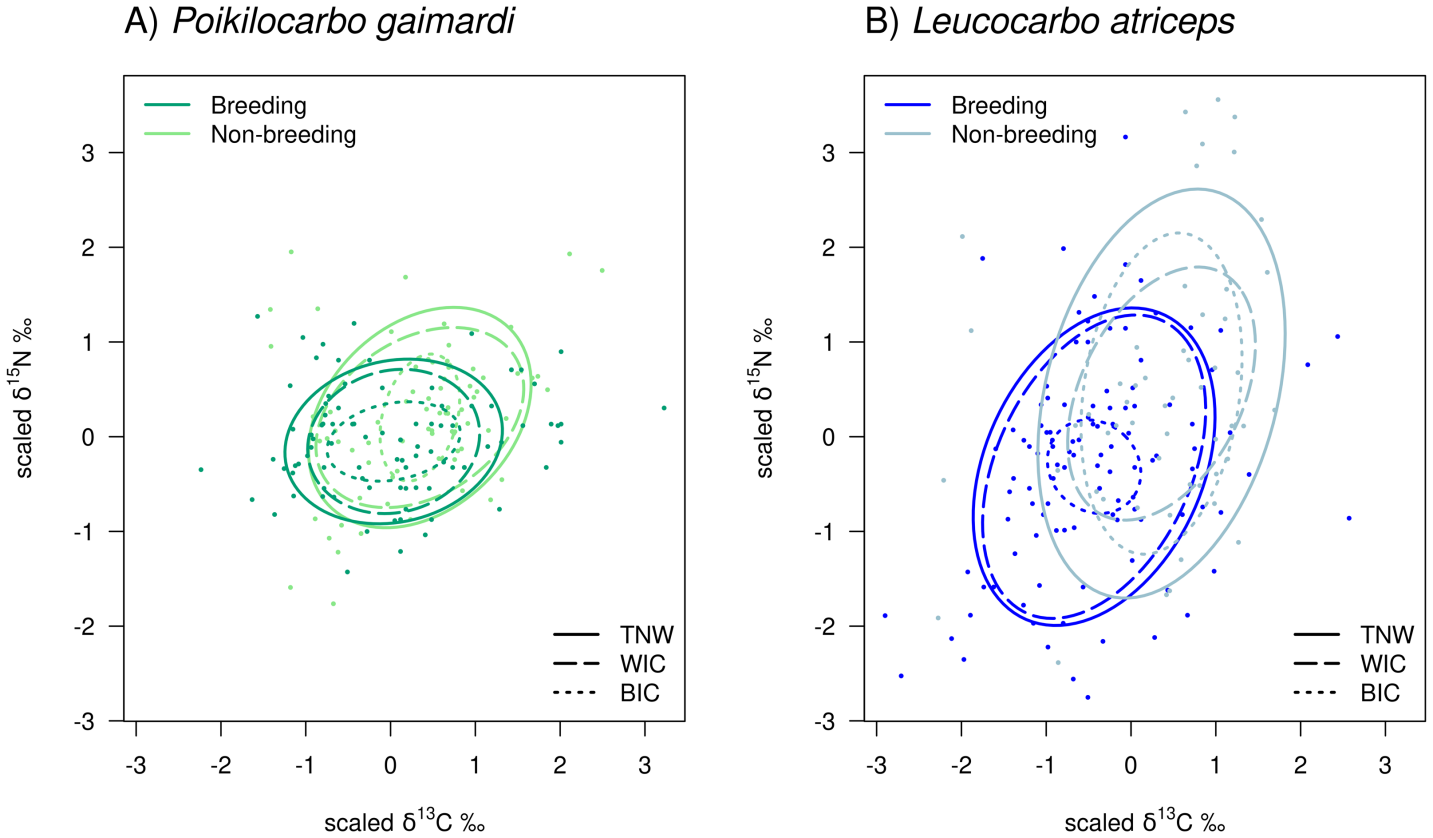
Isotopic niche components of *Poikilocarbo gaimardi* and *Leucocarbo atriceps* during breeding and non-breeding seasons on the Pirén islet, Chile. Total niche width (TNW), within-individual component (WIC), and between-individual component (BIC) for two species, (A) red-legged cormorant (*Poikilocarbo gaimardi*) and (B) imperial shag (*Leucocarbo atriceps*), during both breeding and non-breeding seasons on the Pirén Islet (Los Lagos, Chile). Ellipses represent 67% confidence intervals around the isotopic niche space. The figure was constructed using the posterior means of the model parameters; for clarity, the effects of parameter variability within the posterior distributions are not shown. Axes represent isotopic measures, which have been z-score scaled before analysis.

During the non-breeding season, the *L. atriceps* TNW was substantially (2.2-fold) broader than the *P. gaimardi* TNW (P (TNW_L.atriceps_>TNW_P.gaimardi_)= 0.999; Table 2). This difference was driven by a moderately (24.8%) larger WIC (P (WIC_L.atriceps_>WIC_P.gaimardi_) = 0.707), and by a 5.7-fold larger BIC (P (BIC_L.atriceps_>BIC_P.gaimardi_) = 0.998). Consequently, the *L. atriceps* population displayed a much higher degree of individual specialisation (IS = 0.541), estimated to be 60.1% greater than the *P. gaimardi* one (P (IS_L.atriceps_>IS_P.gaimardi_) = 0.985; Table 2; Fig. 2).

**Table 1:** Stable isotope values (*δ*^13^C and *δ*^15^N) for red-legged cormorant (*Poikilocarbo gaimardi*) and imperial shag (*Leucocarbo atriceps*) across four tissue types. Mean (±SD) stable isotope values of carbon (*δ*^13^C) and nitrogen (*δ*^15^N) for the red-legged cormorant (*Poikilocarbo gaimardi*) and imperial shag (*Leucocarbo atriceps*) across four tissue types (body feathers, primary feathers, plasma, and red blood cells). Analyses were conducted univariately for *δ*^13^C and *δ*^15^N. Different superscript letters denote differences between tissue types within species, and the symbols (triangle, circle) denote differences between species within the same isotope, as determined by the most informative model. Absence of superscript letters or symbols indicates no model achieved an AICw > 0.67.

	*δ*^13^C		*δ*^15^N	
Sample type	*P. gaimardi*	*L. atriceps*	*P. gaimardi*	*L. atriceps*
Body feathers	−14.33 ± 0.245^a^ (*n* = 25)	−14.124 ± 0.256^a^ (*n* = 18)	17.426 ± 0.157^a^ (*n* = 25)	17.426 ± 0.932 (*n* = 18)
Primary feathers	−14.48 ± 0.239^a^ (*n* = 31)	−14.280 ± 0.237^a^ (*n* = 37)	17.683 ± 0.153^b^ (*n* = 31)	17.667 ± 0.728 (*n* = 37)
Plasma	−16.197 ± 0.227^b,^^▴^ (*n* = 56)	−16.714 ± 0.228^b,^^●^ (*n* = 55)	16.993 ± 0.139^c,^^▴^ (*n* = 55)	16.682 ± 0.658^●^ (*n* = 56)
Red blood cells	−15.176 ± 0.227^c,^^▴^ (*n* = 56)	−15.561 ± 0.228^c,^^●^ (*n* = 55)	16.537 ± 0.139^d^ (*n* = 55)	16.575 ± 0.583 (*n* = 56)

**Table 2:** Total niche width, niche components, individual specialisation, and population niche overlap for *Poikilocarbo gaimardi* and *Leucocarbo atriceps* during non-breeding and breeding seasons. Total niche width (TNW), within-individual component (WIC), between-individual component (BIC), individual specialisation (IS = BIC/TNW), and percentage of population niche overlap (% Overlap) are presented for the Red-legged cormorant (*Poikilocarbo gaimardi*) and the Imperial shag (*Leucocarbo atriceps*) during both breeding and non-breeding seasons on the Pirén Islet (Los Lagos, Chile). Values represent the mean ± SE, calculated from the posterior distributions of the analysed data. Different superscript letters (a, b) indicate differences between periods within each species. The symbols (triangle, circle) denote differences between species during the same season. The mean posterior distribution determined this. Absence of superscript letters indicates that no differences were found at *P* > 0.67.

Species	Season	TNW	WIC	BIC	IS=BIC/TNW	% Overlap
*P. gaimardi*	Non-breeding (*n* = 25)	1.39 ± 0.19^a,^^▴^	1.09 ± 0.16^a,^^▴^	0.30 ± 0.14^▴^	0.213 ± 0.08^a,^^▴^	95.7 ± 5.41^a,^^▴^
	Breeding (*n* = 31)	1.09 ± 0.15^b,^^▴^	0.73 ± 0.10^b,^^▴^	0.36 ± 0.14^▴^	0.324 ± 0.09^b,^^▴^	89.6 ± 6.97^b,^^▴^
*L. atriceps*	Non-breeding (*n* = 18)	3.06 ± 0.71^a,^^●^	1.36 ± 0.29^a,^^●^	1.70 ± 0.72^a,^^●^	0.541 ± 0.11^a,^^●^	48.3 ± 9.39^●^
	Breeding (*n* = 37)	2.19 ± 0.23^b,^^●^	1.94 ± 0.22^b,^^●^	0.25 ± 0.11^b,^^●^	0.112 ± 0.05^b,^^●^	43.7 ± 5.93^●^

In contrast, during the breeding season, although *L. atriceps* maintained a 2-fold greater TNW (P (TNW_L.atriceps_>TNW_P.gaimardi_) = 1) and a 2.6-fold higher WIC (P (WIC_L.atriceps_>WIC_P.gaimardi_) = 1) than *P. gaimardi*, but its BIC was 31.2% lower (P (BIC_L.atriceps_<BIC_P.gaimardi_) = 0.750) than in *P. gaimardi* (Table 2; Fig. 2). This shift resulted in opposite changes in individual specialisation, where *P. gaimardi* exhibited an individual specialisation index (IS = 0.324) 31.4% higher than that of *L. atriceps* (IS = 0.112, P (IS_P.gaimardi_>IS_L.atriceps_) = 0.979).

When comparing seasons within species, notable differences in TNW and its components are evident. In *P. gaimardi*, TNW decreased by 21.4% from the non-breeding to the breeding season (P (TNW_breeding_<TNW_non-breeding_) = 0.902). This reduction was mainly driven by a 33.0% decline in the WIC (P (WIC_breeding_<WIC_non-breeding_) = 0.974) despite a 23.1% increase in the BIC (P (BIC_breeding_>BIC_non-breeding_) = 0.632). *L. atriceps* exhibited a similar (28.5%) decrease in TNW over the same season (P (TNW_breeding_<TNW_non-breeding_) = 0.999). However, WIC increased by 43.0% (P (WIC_breeding_>WIC_non-breeding_) = 0.936) in this species, while BIC decreased sharply by 86.7% (P (BIC_breeding_>BIC_non-breeding_) = 0.999), during the breeding season (Table 2; Fig. 2). Along with the overall reduction in TNW, the individual specialisation index (IS) declined by 79.30% in *L. atriceps*, indicating a lower individual specialisation during the breeding season (P (IS_breeding_<IS_non-breeding_) = 0.999). In contrast, *P. gaimardi* showed a slight increase of 52.11% in this index (P (IS_breeding_>IS_non-breeding_) = 0.818).

### Population niche width overlap between species

**Figure 3:**
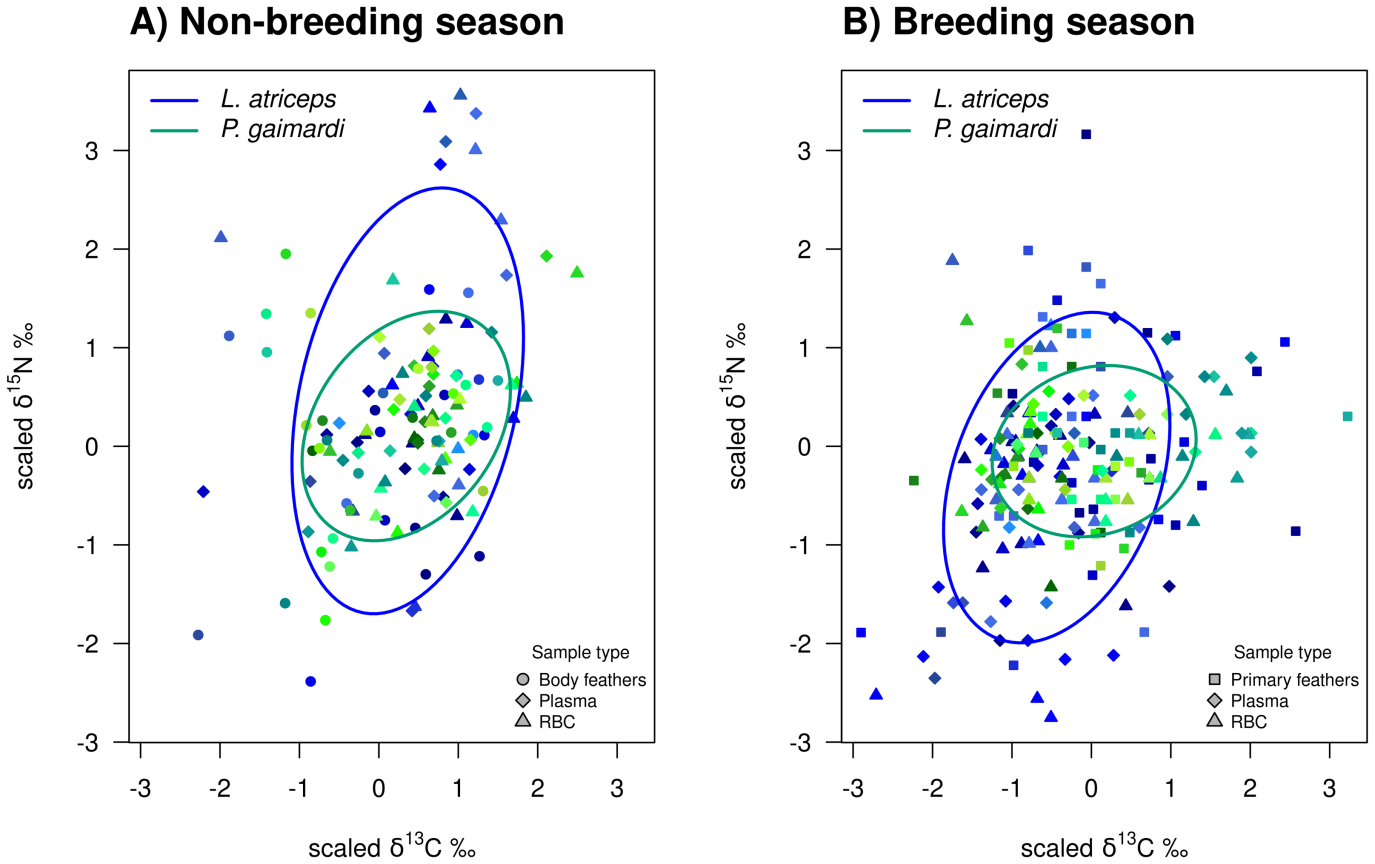
Isotopic niche overlap between *Poikilocarbo gaimardi* and *Leucocarbo atriceps* during breeding and non-breeding periods on the Pirén islet, Chile. Population total niche width (TNW) overlap between red-legged cormorant (*Poikilocarbo gaimardi*) and imperial shag (*Leucocarbo atriceps*) during (A) breeding and (B) non-breeding seasons on the Pirén Islet (Los Lagos, Chile). Ellipses represent 67% confidence levels around the isotopic niche space, based on the posterior means of the model parameters; for clarity, the effects of parameter variability within the posterior distributions are not shown. Isotopic measurements for individual *P. gaimardi* are displayed using a green colour scale, while those for *L. atriceps* are shown in blue. Different symbols indicate distinct sample types. Axes represent isotopic measures, which have been z-score scaled before analysis.

During the non-breeding season, *P. gaimardi* exhibited a very high degree of interspecific niche width overlap (95.7%) with *L. atriceps*. In contrast, only 48.3% of the *L. atriceps’* niche overlapped with that of *P. gaimardi* during this season, consistent with the much broader isotopic niche (TNW) of *L. atriceps* (Fig. 3). During the breeding season, although the interspecific niche width overlap decreased by ∼5–6% for each species, it remained substantially high (89.6%) for *P. gaimardi*, while 43.7% of the *L. atriceps*’s niche overlapped with that of *P. gaimardi* (Fig. 3).

### Ecological opportunity and interspecific competition

**Figure 4:**
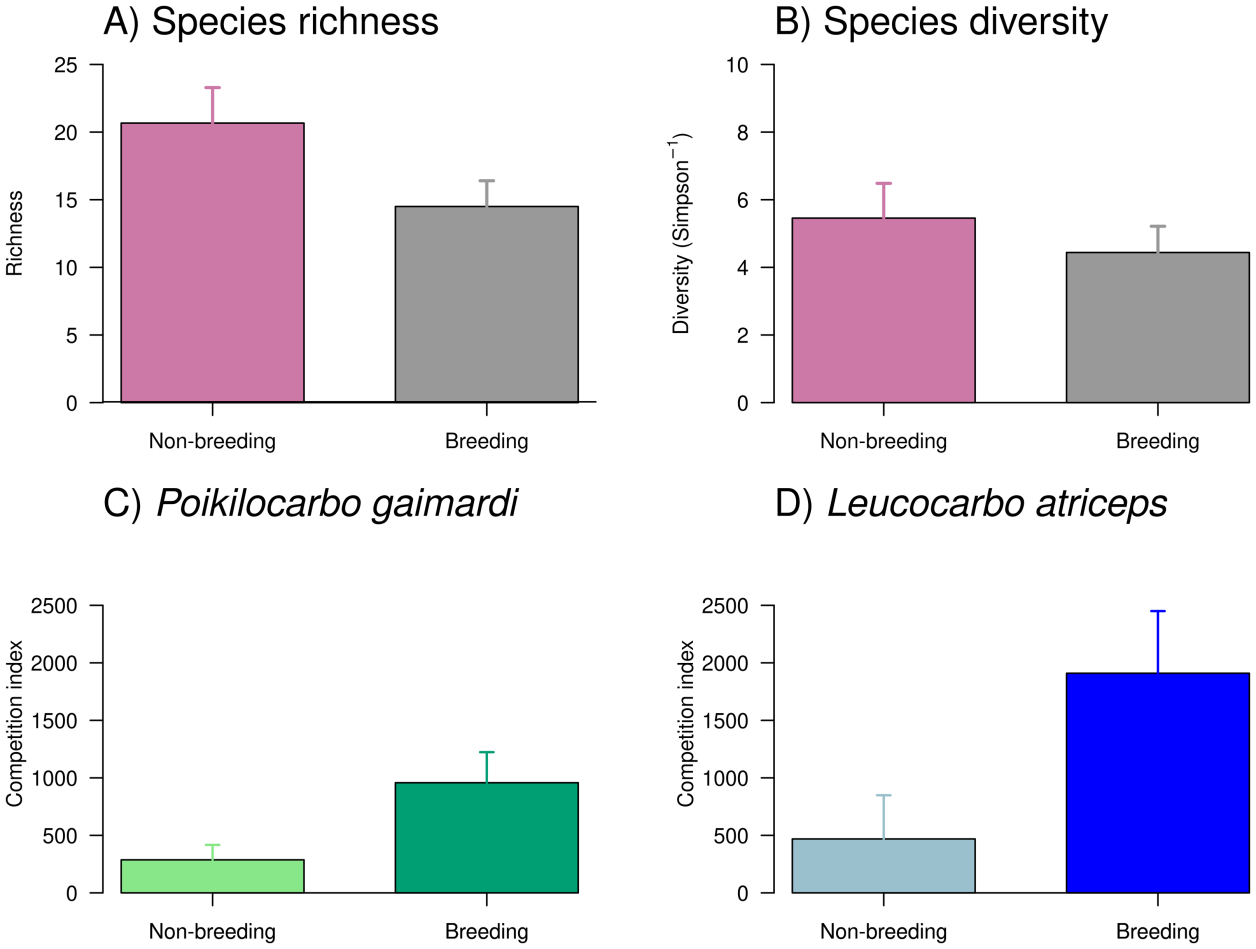
Taxonomic richness, diversity, and competition index of *Poikilocarbo gaimardi* and *Leucocarbo atriceps* during breeding and non-breeding seasons on the Pirén islet, Chile. (A) Taxonomic richness (mean ± standard error) and (B) diversity (inverse Simpson index, mean ± standard error) of taxa identified in seabird pellets collected on the Pirén Islet (Los Lagos, Chile) during breeding and non-breeding seasons in 2020 and 2022. (C) Mean (±espacio standard deviation) competition index for the red-legged cormorant (*Poikilocarbo gaimardi*) and (D) for the imperial shag (*Leucocarbo atriceps*) during non-breeding and breeding seasons from 2020 to 2024 on the Pirén Islet (Los Lagos, Chile).

Overall, the richness and diversity of taxa found in seabird pellets from Pirén Islet were greater during the non-breeding season than during the breeding season (Fig. 4), with species richness increasing by 43% and diversity by 14%. Despite these apparent seasonal shifts, the null models—which assumed no seasonal differences—demonstrated stronger empirical support than seasonal alternatives, suggesting these fluctuations might reflect natural variability rather than systematic seasonal patterns. Main prey (see Table S3 for complete prey composition) included pelagic species such as giant squid *Doryteuthis gahi* (24% and 17% in non-breeding and breeding seasons, respectively) and silverside *Odontesthes regia* (12% and 4%), as well as, benthic species like red octopus *Robsonella fontaniana* (11% and 5%) and blenny *Auchenionchus variolosus* (5% and 7%).

The competition index exhibited a marked seasonal increase during the breeding season and substantial differences between species (Fig. 4, Table S4), where *L. atriceps* showed a seasonal increment of 305% while *P. gaimardi* a somewhat smaller yet considerable increase of ∼221% between seasons. During the non-breeding season, *L. atriceps* exhibited a competition index 75% higher than *P. gaimardi*, a disparity that widened to 121% during the breeding season. Such pattern suggests heightened competitive intensity for *L. atriceps*, particularly during breeding season (Fig. 4, Table S4).

## Discussion

### General patterns

In this study, we aimed to evaluate the mechanisms that facilitate coexistence between two sympatric cormorants (*L. atriceps* and *P. gaimardi* ), focusing on seasonal changes in isotopic niche width, individual specialisation, and interspecific overlap. Our results show that two sympatric and closely related seabird species exhibit substantial seasonal variation in the size and interspecific niche width overlap of their trophic niches, as well as in their niche components and levels of individual specialisation. This variability suggests that these species respond to seasonal changes in prey availability, breeding constraints, and differing dispersal behaviours. These patterns suggest that coexistence is maintained not by a single mechanism but through a combination and alternation of multiple niche partitioning strategies that collectively reduce competition.

The observed tissue-specific isotopic differences (Table 1) reflected variability in both temporal integration windows and discrimination factors (Bearhop et al., 2004; Newsome et al., 2007). *δ*^13^C values increased with turn-over rate in both species, being lowest in plasma (nominal turn-over of 3–7 days) and highest in feathers (nominal turn-over of several weeks). A similar pattern was observed in *δ*^15^N, although red blood cells exhibited higher values than plasma in both species. While a faster turnover rate is expected to reduce isotopic fractionation due to shorter exposure time to chemical reactions, it is also expected to increase sample variability caused by short-term dietary shifts (Cherel & Hobson, 2007; Craig et al., 2015). Not surprisingly, and as commonly reported in other birds, feathers were enriched in both ^13^C and ^15^N when compared to both blood components (Bond & Jones, 2009; Craig et al., 2015).

### Coexistence mechanisms based on niche theory (M1 & M2)

Following classical niche theory, M1—segregation of population niches—posits that population-level niche differentiation would be the primary mechanism enabling coexistence between ecologically similar species (Hardin, 1960; Schoener, 1974). However, our results revealed a considerable interspecific overlap in isotopic niches between the two cormorants, during both seasons. According to classical competitive exclusion theory, such niche overlap would lead to exclusion of one species if resources become limited. However, recent studies show that high niche overlap does not necessarily result in competitive exclusion when mechanisms like individual specialisation and behavioural plasticity buffer interspecific competition (Schirmer et al., 2020; Johnson et al., 2022; Murray et al., 2023). Nonetheless, a subtle segregation in mean isotopic position (primarily in *δ*^13^C) and niche width overlap was observed during the breeding season, revealing that some divergences in prey selection and/or foraging habitats had contributed to mitigating direct competition at the time energetic and nest-guarding constraints compel both species to forage near the colony (Cherel & Hobson, 2007; Phillips et al., 2017; Jessopp et al., 2020).

The considerable niche width overlap we observed on the Pirén islet notably contrasts with the findings of Morgenthaler et al. (2025), which showed great trophic segregation between Atlantic populations of the same two species. These contrasting results may reflect regional differences in ecological opportunity, such as higher prey abundance leading to reduced interspecific competition in our study site. Oceanographic conditions characterise and differentiate both study sites. The Reloncaví Sound exhibits higher, although seasonally variable, productivity related to freshwater inputs (Iriarte, González & Nahuelhual, 2010). In contrast, the Patagonian Shelf is characterised by greater fish diversity associated with the confluence of the warm Brazilian and the cold Malvinas currents (Alemany, Acha & Iribarne, 2009). These geographical disparities underscore the context-dependent nature of coexistence mechanisms, where local abiotic and biotic factors interact with species-specific foraging strategies to shape niche dynamics (Morgenthaler et al., 2025).

It is important to acknowledge that niche segregation can occur across multiple ecological dimensions, and our study examined only *δ*^13^C and *δ*^15^N isotopic dimensions. As shown by Morgenthaler et al. (2025), the probability of detecting interspecific niche segregation increases with the number of dimensions being analysed. The substantial isotopic overlap we observed does not preclude segregation along unmeasured dimensions such as spatial foraging patterns, diving depths, temporal activity, or fine-scale prey preferences. Despite sharing similar isotopic signatures, these species may exploit different microhabitats within the Reloncaví Sound or show temporal segregation in foraging activity, as documented in other sympatric cormorants where species partition time to exploit resources more effectively (Mahendiran, 2016). Nevertheless, we concur that the high abundance of small pelagic fishes in the productive Reloncaví Sound system might be an equally plausible explanation for the observed overlap (Ceia & Ramos, 2015).

While finding marked but contrasting seasonal differences in individual specialisation between the two studied species, our results provided limited support for the notion that individual specialisation (M2—enhanced individual specialisation) plays a significant role in mitigating the effects of enhanced competition (Svanbäck & Bolnick, 2005; Araújo, Bolnick & Layman, 2011). On one hand, the more resident *P. gaimardi* exhibited some increase in individual specialisation during the breeding season, which was explained by a moderate reduction in WIC as individuals differentiated their resource use, possibly to reduce intraspecific competition under heightened breeding constraints (Phillips et al., 2017). On the other hand, the magnitude of this response appeared to be small given the fourfold seasonal increase in *L. atriceps* abundance. The more dispersive *L. atriceps* showed, instead, a decreased individual specialisation during the breeding season, which was explained by a major reduction in BIC that cancelled out a minor decrease in WIC. We speculate this response reflected the drastic reduction in ecological opportunity the species faces during such season, as its foraging range becomes limited by nest-guarding and chick-feeding constraints. This pattern has been also reported in other dispersive seabirds (Harris et al., 2015; Zango et al., 2019), which shift from flexible and extensive foraging ranges during the non-breeding season to colony-constrained central-place foraging during reproduction time (Birt et al., 1987; Dehnhard et al., 2020; Cansse et al., 2024b).

### Mechanisms based on optimal foraging theory (M3 & M4)

Unlike expectations from M3—expansion of the total population niche—and OFT, the total population niche width (TNW) did not increase during the breeding season in either of the two studied species. Instead, a decrease in TNW—particularly pronounced in *L. atriceps*—was observed during this season. This suggests that expanding the trophic population niche to include suboptimal prey was not a strategy used to alleviate the seasonal increase in potential competition, at least in this case study. We speculate that population niche expansion was constrained by limited ecological opportunities around breeding sites, given the relatively short foraging ranges reported for cormorants (*L. triceps*, 10–50 km; *P. gaimardi*, 0.1−4.1 km; Gandini, Frere & Quintana, 2005; Quintana et al., 2010). During the non-breeding season, a noticeable expansion of TNW occurred in both species, but *via* different mechanisms. In the dispersive *L. atriceps*, expansion occurred through increased segregation between individual niches (BIC), following the niche variation hypothesis (Van Valen, 1965), which postulates that the release of competitive constraints permits greater individual diversification in resource use. In contrast, in the resident *P. gaimardi*, it occurred through an expansion of WIC, indicating that individuals broadened their respective dietary niches *via* ‘parallel release’ (Bolnick et al., 2010), where all individuals simultaneously expand their niche widths in response to increased ecological opportunity. These divergent mechanisms highlight how life-history strategies influence species’ responses to ecological opportunities—a crucial consideration for predicting resilience under environmental change.

The WIC of *L. atriceps* increased during the breeding season, as predicted by M4—expansion of the individual niche—, *i.e.,* by the OFT operating at the individual level (Sheppard et al., 2018; Costa-Pereira et al., 2019). The causal relationship between heightened competition and the observed expansion in WIC in *L. atriceps* remains somewhat unclear. While individuals likely expanded their trophic niche to compensate for the diminished availability of optimal or preferred prey (Bolnick et al., 2010), this reduced availability may have resulted from both the seasonally constrained foraging range and increased competition during the breeding season. Regardless, this seasonal expansion of WIC parallels responses observed in other dispersive seabirds facing seasonal spatial constraints, where behavioural plasticity enhances resource exploitation efficiency (Ceia & Ramos, 2015). Additionally, the more dispersive nature of *L. atriceps* means that individuals may exploit completely different prey assemblages across various geographic locations during non-breeding, further contributing to the observed expansion in individual dietary width (Cansse et al., 2024a; Yorio et al., 2020).

Opposing M4 predictions and *L. atriceps’* responses, *P. gaimardi* exhibited a reduction in WIC during the breeding season, which was consistent instead with M2 predictions, *i.e.,* with an individual niche partitioning strategy. These contrasting responses may reflect physiological, anatomical, and behavioural differences between these two species, leading to different levels of individual plasticity—a key trait for withstanding environmental fluctuations and resource depletion (Bolnick et al., 2003; Valladares et al., 2015). The observed patterns may also reflect broader ecological principles: dispersive species, such as *L. atriceps*, leverage plasticity to exploit variable resources (Phillips et al., 2017; Zango et al., 2019), while resident species, like *P. gaimardi*, rely on specialised, consistent foraging tactics (Lewke, 1982; Campana et al., 2022). Additionally, given the *L. atriceps* population is substantially larger than that of *P. gaimardi*, intraspecific competition during the breeding season is likely more intense in *L. atriceps*, which could drive the observed expansion of individual niche width (M4) in this species.

### Implications and conservation considerations

While our stable isotope analysis provided valuable insights into the trophic ecology of *L. atriceps* and *P. gaimardi*, several methodological constraints common to this approach warrant further consideration. First, isotopic niche metrics represent an indirect approach to infer about resource use patterns and rely on several key assumptions. Critical to our case is that isotopic baselines and discrimination factors are spatially and temporally stable (Bearhop et al., 2004; Newsome et al., 2007). Second, stable isotope analysis has a limited ability to resolve fine-scale dietary differences, particularly when prey species share similar isotopic signatures (Newsome, Clementz & Koch, 2010; Phillips et al., 2014). Third, quantifying ecological opportunity and competition indices in marine systems remains inherently challenging due to the dynamic nature of prey availability and the difficulty of directly measuring interspecific interactions in pelagic environments (Harris et al., 2015; Phillips et al., 2017). Hence, our reliance on surrogate indicators may have oversimplified the complex interplay between resource partitioning and competition in this case study.

Finally, we recognise that our analyses are confined to two isotopic axes. Additional niche dimensions—such as breeding phenology, foraging microhabitat or prey-handling behaviour—may reveal further segregation, as demonstrated by Morgenthaler et al. (2025) for *N. brasilianus* and *P. magellanicus*. Future studies, both here and elsewhere, would benefit from integrating direct dietary sampling methods (*e.g.*, regurgitates, GPS tracking, DNA metabarcoding of cloacal swabs) with stable isotope analysis to refine estimates of prey contributions and validate niche partitioning mechanisms (Newsome et al., 2012; Ibarra et al., 2018). While isotopic mixing models (*e.g.*, SIAR, MixSIAR) offer theoretical potential, their application to wild seabirds is severely constrained by the lack of species-specific discrimination factors, which would require extensive captive studies to obtain reliable parameters (Newsome et al., 2012; Ibarra et al., 2018; Morgenthaler et al., 2016; Morgenthaler et al., 2022). Comparative studies of the trophic ecology of allopatric populations would also help determining whether observed niche-width differences are intrinsic or arise from sympatric interactions.

Sampling limitations imposed additional limitations on the current study. The 2023 outbreak of highly pathogenic avian influenza (Azat et al., 2024) abruptly halted our fieldwork, resulting in reduced sample sizes, particularly for *P. gaimardi*, during both the 2023 breeding and non-breeding seasons. Additionally, the logistical difficulties of capturing and handling these birds exceed our expectations, given their sensitivity to disturbance. Furthermore, the roughness of the coastal terrain appears to be higher than previously reported in other colonies of the same two species, particularly for the more elusive *P. gaimardi* (King et al., 1994; Morgenthaler et al., 2025). Larger, multi-year datasets would strengthen the robustness of individual specialisation metrics and improve statistical power to detect subtle seasonal shifts (Bolnick et al., 2003; Ceia & Ramos, 2015).

Potential effects of interanual oceanographic variability might have affected our study as our sampling occurred during the transition from the prolonged 2020–2022 La Niña to El Niño conditions affecting the Chilean coast during late 2022/early 2023. Extended La Niña enhanced productivity may have eased competition, whereas emerging El Niño conditions could have intensified it. Multi-year sampling across ENSO phases is therefore needed to fully characterise how climatic oscillations influence niche dynamics.

Despite the ecological and economic relevance of the SE Pacific coastline, few ecological studies have addressed the trophic ecology and niche dynamics of coastal seabirds, such as cormorants. Even fewer have focused on individual specialisation, whose understanding may be critical to enhancing conservation strategies for marine birds, given its role in buffering populations against environmental change (Bolnick et al., 2011; Valladares et al., 2015; Manlick, Maldonado & Newsome, 2021). Our findings demonstrate that the coexistence of sympatric cormorants hinges not on broad population-level segregation but on nuanced individual strategies, underscoring the importance of preserving heterogeneous foraging habitats to sustain ecological opportunity. This consideration may be especially important for *P. gaimardi*, which is currently classified as “Near Threatened” according to the IUCN Red List (BirdLife International, 2021). The species’ narrower individual niches and higher isotopic niche width overlap, compared with *L. atriceps* suggest competitive vulnerability. Improving knowledge on resource use, protecting prey availability and environmental quality in foraging zones, as well as reducing anthropogenic pressures over breeding sites, are the most urgent tasks here (Furness et al., 2012; Thaxter et al., 2012; Soldatini et al., 2015).

## Conclusions

Our results indicate that coexistence between dispersive *L. atriceps* and the resident *P. gaimardi* on the Pirén Islet is sustained by a dynamic interplay of niche width partitioning and resource-use strategies, which shift according to season and ecological conditions. The dispersive species consistently exhibits a broader population niche than the resident, particularly during the non-breeding season when ecological opportunity is greater and competition is reduced. Despite a high degree of niche width overlap between the species, the dispersive cormorant’s ability to exploit a wider range of resources, likely due to its mobility, appears to buffer direct interspecific competition.

The evidence for individual specialisation as a mechanism underpinning coexistence between these cormorant species is suggestive but not unequivocal, since the width of the isotopic niche differed but the overall isotopic niche overlapped. While patterns of individual specialisation are observed—such as higher specialisation in *L. atriceps* during the non-breeding season and a modest increase in *P. gaimardi* during breeding—these trends are not sufficiently strong or consistent to be considered definitive proof. Instead, the results point to a more nuanced scenario in which individual specialisation may contribute to coexistence, but its role is likely context-dependent and intertwined with other ecological processes.

In summary, the coexistence of these closely related cormorant species cannot be attributed to a single mechanism. Rather, it results from the combined effects of partial niche segregation, varying levels of individual specialisation, and context-dependent responses to competition and ecological opportunity. Crucially, divergent life-history strategies also play a key role, highlighting the need to consider both intra- and interspecific variation when analysing the ecological processes that sustain biodiversity in complex communities.

##  Supplemental Information

10.7717/peerj.20384/supp-1 Supplemental Information 1 Mean and standard deviation of raw isotopic data (*δ*^13^C and *δ*^15^N) for two sympatric cormorant species captured on the Pirén Islet during 2022 and 2023 Mean and standard deviation of the raw isotopic data (*δ*^13^C and *δ*^15^N) from the two sympatric cormorant species, red-legged cormorant (*Poikilocarbo gaimardi*) and imperial shag (*Leucocarbo atriceps*), captured on the Pirén Islet during 2022 and 2023, used for standardised isotopic values within tissues across species.

10.7717/peerj.20384/supp-2 Supplemental Information 2 Morphometric measurements of red-legged cormorant (*Poikilocarbo gaimardi*) and imperial shag (*Leucocarbo atriceps*) captured on the Pirén Islet during 2022–2023 Values are presented as mean ± standard error. Different superscript letters denote differences between species as determined by the informative model. Absence of superscript letters indicates no model achieved an AICw > 0.67.

10.7717/peerj.20384/supp-3 Supplemental Information 3 Median composition of prey taxa identified in seabird pellets collected on the Pirén Islet during breeding and non-breeding periods of 2020 and 2022 Median composition of prey taxa identified in seabird pellets collected on the Pir é n Islet during the breeding and non-breeding period of years 2020 and 2022. Medians and 95% credibility intervals computed using Bayesian zero-and-one inflated Dirichlet regression as implemented in the R package ‘zoid’ (Ward et al. 2024).

10.7717/peerj.20384/supp-4 Supplemental Information 4 Seasonal abundance patterns of seabirds on the Pirén Islet (2021–2024) Mean abundance (±standard deviation) of seabirds recorded during the breeding season (October–January) and non-breeding season (May–August) censuses conducted between 2021 and 2024 on Pirén Islet. Values represent average abundances calculated across all census dates within each seasonal period. ”No data” indicates no observations were recorded for the respective species during the given season. The nomenclature of cormorant species follows the taxonomic revision by Kennedy & Spencer (2014).

## Additional Information and Declarations

### Field Study Permissions

The following information was supplied relating to field study approvals (*i.e.*, approving body and any reference numbers): Field experiments were approved by Resolution No. 7850 from the Servicio Agrícola y Ganadero (SAG), Chile.

### Data Availability

The following information was supplied regarding data availability: The data supporting this study’s findings are available at Figshare: Piriz, Gabriela (2025). Morphological and stable isotope data from sympatric cormorant species in southern Chile. figshare. Dataset. https://doi.org/10.6084/m9.figshare.29526404.v1.
