# Supplementary material for: Individual and population variation in isotopic niche between two sympatric cormorant species
Source: PeerJ. 2026 Jan 12;14:e20384. doi: 10.7717/peerj.20384 (PMC12805912; doi:10.7717/peerj.20384)
Supplement: Supplementary file 1 [file peerj-14-20384-s001.odt]

Table S1. Mean and standard deviation of raw isotopic data (δ¹³C and δ¹⁵N) for two sympatric cormorant species captured on the Pirén Islet during 2022 and 2023.

Mean and standard deviation of the raw isotopic data (δ¹³C and δ¹⁵N) from the two sympatric cormorant species, red-legged cormorant (Poikilocarbo gaimardi) and imperial shag (Leucocarbo atriceps), captured on the Pirén Islet during 2022 and 2023, used for standardised isotopic values within tissues across species.

| Sample type | δ13C | δ15N |
| --- | --- | --- |
| Body feathers (n= 43) | -13.94 ± 0.919 | 17.49 ± 0.825 |
| Primary feathers (n= 68) | -14.66 ± 0.547 | 17.62 ± 0.594 |
| Plasma (n= 111) | -16.52 ± 0.854 | 16.83 ± 0.523 |
| Red blood cells (n= 111) | -15.43 ± 0.725 | 16.55 ± 0.453 |
