# Supplementary material for: Individual and population variation in isotopic niche between two sympatric cormorant species
Source: PeerJ. 2026 Jan 12;14:e20384. doi: 10.7717/peerj.20384 (PMC12805912; doi:10.7717/peerj.20384)
Supplement: Supplementary file 2 [file peerj-14-20384-s002.odt]

Table S2. Morphometric measurements of Red-legged Cormorant (Poikilocarbo gaimardi) and Imperial Shag (Leucocarbo atriceps) captured on Pirén Islet during 2022–2023. 

Morphometric measurements of Red-legged Cormorant (Poikilocarbo gaimardi) and Imperial Shag (Leucocarbo atriceps) captured on Pirén Islet during 2022–2023. Values are presented as mean ± standard error. Different superscript letters denote differences between species, as determined by the most informative model.

| Morphometric measure | Poikilocarbo gaimardi (n=56) | Leucocarbo atriceps (n=55) |
| --- | --- | --- |
| Mass (kg) | 1.55 ± 0.022a | 2.12 ± 0.031b |
| Wing length (cm) | 25.75 ± 0.257a | 27.31 ± 0.259b |
| Bill length (cm) | 8.91 ± 0.473 | 8.95 ± 0.479 |
| Bill width (cm) | 1.28 ± 0.017a | 1.59 ± 0.021b |
| Tail length (cm) | 13.71 ± 0.137a | 14.81 ± 0.149b |
| Leg length (cm) | 14.35 ± 0.047a | 16.19 ± 0.091b |
