# Supplementary material for: Individual and population variation in isotopic niche between two sympatric cormorant species
Source: PeerJ. 2026 Jan 12;14:e20384. doi: 10.7717/peerj.20384 (PMC12805912; doi:10.7717/peerj.20384)
Supplement: Supplementary file 3 [file peerj-14-20384-s003.odt]

Table S3. Median composition of prey taxa identified in seabird pellets collected on the Pirén Islet during breeding and non-breeding periods of 2020 and 2022. 

Median composition of prey taxa identified in seabird pellets collected on the Pirén Islet during the breeding and non-breeding period of the years 2020 and 2022. Medians and 95% credibility intervals computed using Bayesian zero-and-one inflated Dirichlet regression as implemented in the R package ‘zoid’ (Ward et al. 2024).

| Feeding habit | Taxon | Non-breeding | Breeding |
| --- | --- | --- | --- |
| Pelagic | Doryteuthis gahi | 24.03 (17.6 – 30.92) | 16.54 (10.79 – 23.63) |
| Odontesthes regia | 12.15 (7.08 – 18.14) | 3.73 (1.14 – 8.07) |
| Clupeidae | 8.48 (4.49 – 13.53) | 7.76 (3.43 – 13.8) |
| Engraulis ringens | 1.66 (0.48 – 4.03) | 7.37 (3.54 – 12.73) |
| Normanichthys crockeri | 1.27 (0.27 – 3.37) | 6.6 (2.7 – 12.31) |
| Galaxiidae | 0.47 (0.08 – 1.55) | 0.22 (0 – 1.22) |
| Mugilidae | 0.47 (0.07 – 1.56) | 0.22 (0 – 1.23) |
| Benthic | Robsonella fontaniana | 11.23 (6.38 – 17.18) | 5.1 (2.28 – 9.63) |
| Auchenionchus variolosus | 5.46 (3.04 – 8.97) | 7.21 (3.03 – 13.05) |
| Enteroctopus megalocyathus | 4.1 (2.72 – 6.41) | 4.13 (1.8 – 7.89) |
| Sindoscopus australis | 3.7 (1.19 – 7.62) | 2.75 (0.59 – 6.73) |
| Nototheniidae | 3.5 (1.22 – 7.09) | 4.65 (2.24 – 8.55) |
| Aphos porosus | 3.47 (1.09 – 7.33) | 5.02 (2.25 – 9.32) |
| Patagonotothen | 1.82 (0.62 – 4.13) | 3.06 (0.91 – 6.91) |
| Hypsoblennius sordidus | 1.6 (0.45 – 3.85) | 2.67 (0.64 – 6.47) |
| Gobiesox marmoratus | 1.59 (0.73 – 3.23) | 0.27 (0 – 1.46) |
| Octopoda | 1.23 (0.48 – 2.73) | 0.26 (0 – 1.38) |
| Grimothea gregaria | 0.97 (0.29 – 2.5) | 3.3 (1.36 – 6.64) |
| Genypterus blacodes | 0.62 (0.1 – 2.02) | 2.32 (0.47 – 5.84) |
| Myxodes viridis | 0.56 (0.09 – 1.86) | 1.29 (0.25 – 3.72) |
| Iluocoetes | 0.55 (0.09 – 1.77) | 1.49 (0.25 – 4.16) |
| Bovichtidae | 0.47 (0.07 – 1.55) | 0.22 (0 – 1.23) |
| Congiopodidae | 0.47 (0.07 – 1.56) | 0.22 (0 – 1.25) |
| Decapoda | 0.47 (0.07 – 1.56) | 0.22 (0 – 1.2) |
|  | Myopsida | 0.12 (0 – 0.68) |
| Paralichthyidae | 0.47 (0.07 – 1.53) | 0.22 (0 – 1.23) |
| Agonopsis chiloense | 0.46 (0.07 – 1.54) | 0.22 (0 – 1.21) |
| Trematomus | 0.46 (0.07 – 1.54) | 0.22 (0 – 1.23) |
| Malacostraca | 0.23 (0.01 – 1.04) | 0.18 (0 – 1.07) |
| Cottoperca | 0.2 (0.01 – 1) | 1.42 (0.22 – 4.03) |
| Genypterus chilensis | 0.2 (0 – 0.99) | 0.85 (0.1 – 2.81) |
| Myopsida | 0.12 (0 – 0.68) | 0.14 (0 – 0.87) |
| Benthic-pelagic | Sebastes oculatus | 4.27 (1.54 – 8.48) | 4.95 (1.59 – 10.08) |
| Merluccius australis | 0.65 (0.09 – 2.09) | 0.23 (0 – 1.26) |
| Merluccius gayi | 0.48 (0.22 – 0.87) | 0.46 (0.18 – 0.93) |
| Pinguipes chilensis | 0.48 (0.07 – 1.54) | 0.22 (0 – 1.24) |
| Sebastes capensis | 0.47 (0.07 – 1.59) | 0.22 (0 – 1.25) |
| Eleginops maclovinus | 0.25 (0.01 – 1.19) | 2.26 (0.43 – 5.74) |
| Macruronus magellanicus | 0.12 (0 – 0.69) | 0.14 (0 – 0.9) |
| Salilota australis | 0.12 (0 – 0.69) | 0.14 (0 – 0.89) |

Ward EJ, Jensen AJ, Kelly RP, Shelton AO, Anderson EA, Satterthwaite WH (2024) zoid: Bayesian zero-and-one inflated Dirichlet regression modelling for compositional data.
