# Supplementary material for: Individual and population variation in isotopic niche between two sympatric cormorant species
Source: PeerJ. 2026 Jan 12;14:e20384. doi: 10.7717/peerj.20384 (PMC12805912; doi:10.7717/peerj.20384)
Supplement: Supplementary file 4 [file peerj-14-20384-s004.odt]

Table S4. Seasonal abundance patterns of seabirds on the Pirén Islet (2021–2024). 

Mean abundance (± standard deviation) of seabirds recorded during the breeding season (October–January) and non-breeding season (May–August) censuses conducted between 2021 and 2024 on Pirén Islet. Values represent average abundances calculated across all census dates within each seasonal period. "No data" indicates no observations were recorded for the respective species during the given season. The nomenclature of cormorant species follows the taxonomic revision by Kennedy & Spencer (2014).

| Species | Orden | Breeding abundance | Non-breeding abundance |
| --- | --- | --- | --- |
| Chroicocephalus maculipennis | Charadriiformes | No data | 1 ± NA |
| Haematopus ater | Charadriiformes | 19.27 ± 4.34 | 16.14 ± 5.96 |
| Haematopus palliatus | Charadriiformes | 3 ± 2 | 3 ± 2 |
| Larus dominicanus | Charadriiformes | 2227.45 ± 755.39 | 527.57 ± 600.61 |
| Leucophaeus scoresbii | Charadriiformes | 45.91 ± 20.47 | 51.71 ± 25.19 |
| Sterna hirundinacea | Charadriiformes | 2.67 ± 1.53 | 2 ± NA |
| Vanellus chilensis | Charadriiformes | No data | 21 ± NA |
| Egretta thula | Pelecaniformes | 12 ± 9.57 | No data |
| Pelecanus thagus | Pelecaniformes | 355.09 ± 218.43 | 24 ± 39.5 |
| Theristicus melanopis | Pelecaniformes | 2.17 ± 0.98 | 2.67 ± 0.58 |
| Spheniscus humboldti | Sphenisciformes | 1.5 ± 0.71 | No data |
| Spheniscus magellanicus | Sphenisciformes | 9 ± 9.08 | No data |
| Leucocarbo atriceps | Suliformes | 1560.78 ± 429.63 | 394.14 ± 386.08 |
| Leucocarbo bougainvillii | Suliformes | 14.8 ± 14.75 | 4.8 ± 3.56 |
| Leucocarbo magellanicus | Suliformes | 47.55 ± 16.45 | 16.14 ± 19.78 |
| Nannopterum brasilianus | Suliformes | 215.83 ± 129.44 | 8.57 ± 8.64 |
| Poikilocarbo gaimardi | Suliformes | 298.02 ± 135.19 | 136.57 ± 54.29 |
| Sula variegata | Suliformes | 142.73 ± 74.08 | 49.29 ± 44.24 |
